# An Amphiprotic Novel Chitosanase from *Bacillus mycoides* and Its Application in the Production of Chitooligomers with Their Antioxidant and Anti-Inflammatory Evaluation

**DOI:** 10.3390/ijms17081302

**Published:** 2016-08-10

**Authors:** Tzu-Wen Liang, Wei-Ting Chen, Zhi-Hu Lin, Yao-Haur Kuo, Anh Dzung Nguyen, Po-Shen Pan, San-Lang Wang

**Affiliations:** 1 Life Science Development Center, Tamkang University, New Taipei City 25137, Taiwan; ltw27@ms55.hinet.net; 2 Department of Chemistry, Tamkang University, New Taipei City 25137, Taiwan; gash3520@gmail.com (W.-T.C.); popan@mail.tku.edu.tw (P.-S.P.); 3 Division of Chinese Materia Medica Development, National Research Institute of Chinese Medicine, Taipei 11221, Taiwan; tiger77749@gmail.com (Z.-H.L.); kuoyh@nricm.edu.tw (Y.-H.K.); 4 Institute of Biotechnology and Environment, Tay Nguyen University, Buon Ma Thuot 630000, Vietnam; nadzungtaynguyenuni@yahoo.com.vn

**Keywords:** chitosanase, amphiprotic, squid pen, *Bacillus mycoides*, chitooligomers, antioxidant

## Abstract

The objectives of this investigation were to produce a novel chitosanase for application in industries and waste treatment. The transformation of chitinous biowaste into valuable bioactive chitooligomers (COS) is one of the most exciting applications of chitosanase. An amphiprotic novel chitosanase from *Bacillus* *mycoides* TKU038 using squid pen powder (SPP)-containing medium was retrieved from a Taiwan soil sample, which was purified by column chromatography, and characterized by biochemical protocol. Extracellular chitosanase (CS038) was purified to 130-fold with a 35% yield, and its molecular mass was roughly 48 kDa. CS038 was stable over a wide range of pH values (4–10) at 50 °C and exhibited an optimal temperature of 50 °C. Interestingly, the optimum pH values were estimated as 6 and 10, whereas CS038 exhibited chitosan-degrading activity (100% and 94%, respectively). CS038 had *K*_m_ and *V*_max_ values of 0.098 mg/mL and 1.336 U/min, separately, using different concentrations of water-soluble chitosan. A combination of the high performance liquid chromatography (HPLC) and matrix-assisted laser desorption ionization-time of flight (MALDI-TOF) mass spectrometer data revealed that the chitosan oligosaccharides obtained from the hydrolysis of chitosan by CS038 comprise oligomers with multiple degrees of polymerization (DP), varying from 3–9, as well as CS038 in an endolytic fashion. The TKU038 culture supernatant and COS mixture exhibited 2,2-diphenyl-1-picrylhydrazyl (DPPH) scavenging activities. The COS activities were dose dependent and correlated to their DP. The COS with high DP exhibited enhanced DPPH radical scavenging capability compared with COS with low DP. Furthermore, the COS exhibited inhibitory behavior on nitric oxide (NO) production in murine RAW 264.7 macrophage cells, which was induced by *Escherichia coli* O111 lipopolysaccharide (LPS). The COS with low DP possesses a more potent anti-inflammatory capability to decrease NO production (IC_50_, 76.27 ± 1.49 µg/mL) than that of COS with high DP (IC_50_, 82.65 ± 1.18 µg/mL). Given its effectiveness in production and purification, acidophilic and alkalophilic properties, stability over ranges of pH values, ability to generate COS, antioxidant activity, and anti-inflammatory, CS038 has potential applications in SPP waste treatment and industries for COS production as a medical prebiotic.

## 1. Introduction

Chitin is one of the most abundant carbohydrate polymers in nature, and chitin can produce chitosan by full or partial deacetylation. Each year, nearly 80,000 metric tons of chitin are generated from marine wastes, including shrimps, crabs, and squids [1]. Chitooligomers (COS) are degraded compounds of chitosan. Chitosan with an average molecular weight of less than 3900 Da or degrees of polymerization of less than 20 are so-called COS [2]. Several studies have previously attracted interest in converting chitosan into COSs due to high water-solubility, low viscosity, and excellent biological properties of COSs [3,4,5,6,7]. In order to acquire these depolymerized molecules, two primary strategies have been developed: chemical and enzymatic. Enzymatic depolymerizing of chitosan is very useful and a more environmentally friendly process for producing COS with various degrees of polymerization (DP). Thus, acquiring an efficient protocol for chitosanase production and the transformation of chitosan into bioactive COS would be vastly desirable for efficiently generation of these oligomeric chitosans.

Chitosanases have been discovered in richness in diversity of bacteria, including *Bacillus* sp. [8,9,10,11], *Serratia* sp. [12], *Janthinobacterium* sp. [13], *Paenibacillus* sp. [14], *Pseudomonas* sp*.* [15], *Acinetobacter* sp. [16] and *Streptomyces* sp. [17]. However, most chitosanases have optimum pH values of approximately 5–6 and weak acidic conditions. In addition, most chitosanases are unstable under acidic or alkaline condition, thus limiting their application, bioconversion, and utilization. Therefore, screening of new chitosanases that are stable under acidic or alkaline conditions similar to those of soil and marine environments is required for extending the application and utilization of chitosanase in industries and for waste treatment.

In the effort to screen chitosanolytic enzymes that are suited for transforming chitosan into large size-oligomeric chitosans, a novel bacterial strain with chitosan degrading capability was obtained. A *Bacillus* *mycoides* strain, TKU038, which was able to utilize squid pen powder (SPP) to generate chitosanase with a satisfactory yield was identified from soil samples. The biochemical features of this chitosanase were fully illustrated after it was purified. The chitosanase was active over ranges of pH values and possessed increased catalytic activity under weak acidic and alkaline conditions compared with previously isolated chitosanases. Furthermore, the applications of the endo-type TKU038 chitosanase in functional chitooligomer generation were also studied. Subsequently, we investigated the antioxidant activity of COS against 2,2-diphenyl-1-picrylhydrazyl (DPPH). The effect of DP on DPPH radical scavenging activity was discussed to identify the optimal DP range with this method. The inhibitory profiles of all COSs on the generation of nitric oxide (NO) stimulated by lipopolysaccharide (LPS) in RAW 264.7 macrophage cells was also evaluated.

## 2. Results and Discussion

### 2.1. Screening and Identification of a Chitosanase-Producing Strain

Over 200 bacterial strains gathered from a selection of cities in Taiwan were cultivated in SPP medium at 37 °C and 150 rpm for three days. Among them, strain TKU038 exhibited strong chitosan degrading capability and was chosen for more in-depth inspection. Based on morphological and biochemical studies, and 16S rDNA sequences [18], the strain was confirmed as *Bacillus* sp. Based on the Analytical Profile Index (API) identification [18], strain TKU038 was the closest to *B.* *mycoides* with 88.5% similarity. Hence, the isolate was identified as *B.* *mycoides*.

### 2.2. Production and Purification of Chitosanase

Fifty milliliters of basal medium (0.1% K_2_HPO_4_ and 0.05% MgSO_4_·7H_2_O, pH 7) containing 0.5% SPP was the most suitable medium for the production of chitosanase by strain TKU038 at 25 °C. The highest chitosanase activity of *B.* *mycoides* TKU038 was detected in the culture on the fourth day of bacterial growth. The culture supernatant exerted strong chitosan degrading activities. The results suggested that the chitosanase from *B.* *mycoides* TKU038 may be secreted extracellularly.

**Figure 1:**
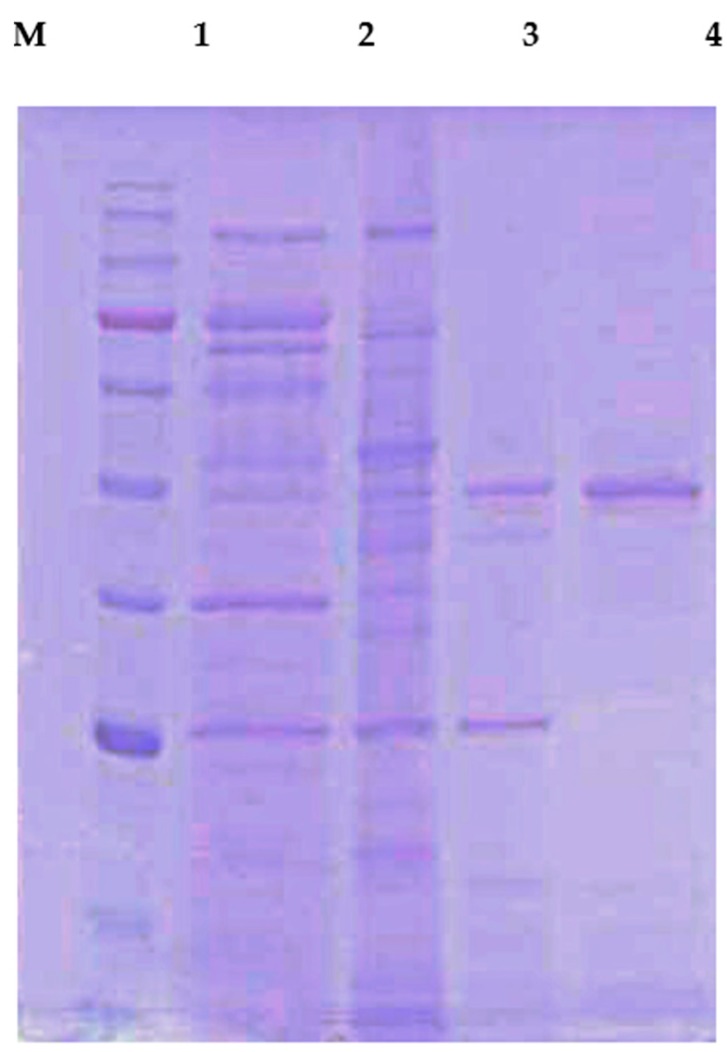
SDS-PAGE analysis of CS038. Lanes: **M** molecular markers (180, 130, 100, 75, 63, 48, 35, 28, 17 and 10 kDa); **1** culture supernatant; **2** crude enzyme; **3** adsorbed chitosanase fractions after DEAE-Sepharose CL-6B chromatography; **4** adsorbed chitosanase fractions after Macro-prep DEAE chromatography.

Extracellular chitosanase was purified from the cell free culture filtrate of *B.* *mycoides* TKU038 using a series of purification procedures. A summary of the CS038 purification is illustrated in Table 1. CS038 was purified to 130-fold with a recovery yield of 35% and a specific activity of 20.82 U/mg. The molecular mass of CS038 was approximately 48 kDa as confirmed by sodium dodesyl sulphate-polyacrylamide gel electrophoresis (SDS-PAGE) (Figure 1), which agreed with the gel-filtration chromatography results. Its molecular mass was similar to chitosanase from *B. cereus* [8,18,19,20,21], as shown in Table 2. Chitosanases from various microbes have been discovered, including bacteria, actinomyces, and fungi, especially *Bacillus* species [8,18,19,20,21,22,23,24,25,26,27,28]. Bacteria produce chitosanase more easily and rapid than fungi in large-scale fermentation systems. However, regarding chitosanase from *Bacillus* species, no study has reported on chitosanase produced by *B.* *mycoides*. This is the first report of the production of chitosanase from *B.* *mycoides*.

### 2.3. Identification of CS038 by LC-MS/MS Analysis

To identify the CS038 that appeared as a prominent 48-kDa band via SDS-PAGE, the band was excised and analyzed after tryptic digestion. The SDS-PAGE gel band was subjected to electrospray tandem mass spectrometry analysis. The fragment spectra were subjected to a NCBI non-redundant protein database search. As shown in Table 3, the spectra of CS038 matched eight tryptic peptides that were identical to the chitosanase from *B. cereus* (GenBank accession number gi446936339) with 54% sequence coverage, and the other remaining peptides were unmatched. The peptide sequences indicate that CS038 belongs to the family 8 glycosyl hydrosylase based on the amino acid sequence similarity of the cited GH-8 enzymes from *B. cereus*.

### 2.4. Effect of pH and Temperature on the Activity and Stability of CS038

**Figure 2:**
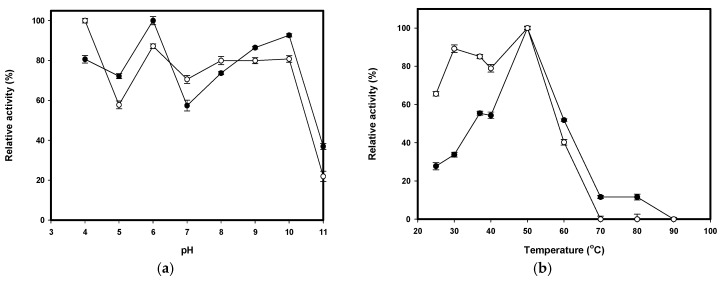
Effects of pH (**a**) and temperature (**b**) on CS038 chitosanase activity (●) and stability (○).

Enzyme activity and stability were markedly affected by pH and temperature. The effect of pH and temperature on CS038 was investigated and is presented in Figure 2. CS038 was active over a wide range of pH values. Comparing to previously isolated chitosanases (Table 2), it possesses higher catalytic activity either under weak acidic (pH 6) or alkaline (pH 10) conditions. Similar results of dual optimum pH were also found in those of *Bacillus cereus* TKU030 chitosanase (pH 4 and 7) [31] and *Mycobacter* AL-1 chitosanase (pH 5.0 and 6.8) [32]. CS038 was stable over a broad range of pH values from 4–10, as shown in Figure 2a. Further, the stability over a broad range of pH values may be due to the reversible denaturation of the protein such that there is no effect on the activity of the enzyme at different pH values. Furthermore, CS038 was found to be more stable in acidic and alkaline media than some of the chitosanases shown in Table 2. For the effect of temperature on activity, CS038 was active over the range of 37 to 60 °C and was the most active at 50 °C (Figure 2b). The effect of temperature on stability was investigated by measuring residual activity after pre-incubating the enzyme at different temperatures for 60 min. Greater than 65% of the initial activity was retained after incubation at 25, 30, 37, 40, and 50 °C (Figure 2b). Approximately 40% of the residual activity could be detected after incubation at 60 °C, but the enzyme was completely inactivated at 70 °C (Figure 2b). The optimal temperature and stability of CS038 was similar to those of the chitosanase from *B. cereus* TKU031 [19], *B. cereus* TKU033 [20], and *B. cereus* TKU034 [21], as shown in Table 2. Many industrial processes are performed at extreme pH values (either acidic or alkaline) and elevated temperatures; thus, the enzyme must suit the process requirements. In addition, higher temperatures (50–60 °C) increase the solubility of polymeric substrates, such as carbohydrates, thereby improving their mechanical handling characteristics and rendering them more amenable to enzymatic attack. Given its acidophilic and alkalophilic nature, tolerance to a broad range of pH values, high optimum temperature, and stability, CS038 is a novel chitosanase compared with those previously reported in *Bacillus* sp.

### 2.5. Substrate Specificity and Kinetic Parameters

For the substrate specificity of purified CS038, chitin and chitosan with DD ranging from 60% to 98% were used as substrates (table not shown). The highest activity was observed in the presence of water-soluble chitosan; however, some detectable activity was observed against other substrates. However, these activities are not considered to be significant compared with chitosanase activity. CS038 showed no activity towards colloidal chitin, shrimp shells, shrimp heads and chitosan with 60% DD, but decomposed 73% DD chitosan at 34% of the activity of water-soluble chitosan.

The kinetic constants (*K*_m_) and (*V*_max_) of CS038 were determined to be 0.098 mg/mL and 1.336 U/min mg, respectively, using a Lineweaver–Burk plot with different concentrations of water-soluble chitosan (0.005%–0.15% (*w*/*v*)). The *Km* value was lower than that of the other chitosanases, such as 0.63 mg/mL from *B. criculans* MH-K1 [28] and 2.1 mg/mL from *Streptomyces griseus* [29], suggesting that the affinity for the substrate of CS038 obtained in this study was better than that of chitosanases from other microorganisms.

### 2.6. Effects of Metal Ions

The influence of metal ions on the activities of CS038 was studied, as shown in Table 4. The activity was inhibited by 5 mM of Cu^2+^, Ba^2+^, Zn^2+^, Fe^2+^, and Mn^2+^. As a chelator in the reaction mixture, ethylendiaminetetraacetic acid (EDTA) also decreased enzyme activity (Table 4) to levels similar to those of the chitosanases from *B. cereus* TKU031 [19] and *B. cereus* TKU034 [21]. Cu ions catalyse the auto-oxidation of cysteines to form intra molecular disulphide bridges or sulphenic acid [31]. Interestingly, the activity of CS038 was nearly unaffected by Na^+^, Mg^2+^, and Ca^2+^, which is similar to the chitosanase from *B. cereus* TKU030 [31]. However, unlike *B. cereus* TKU030, CS038 was inhibited by phenylmethanesulfonyl fluoride (PMSF). These results provide an insight of which metals or chemicals should be selected when specific industrial applications are needed.

Purified CS038 was pre-incubated with the various reagents at 25 °C for 30 min, and residual chitosanase activity was determined as described in the text. One hundred percent was assigned to the activity in the absence of reagents. The relative activity of the chitosanase: 100% = 2.47 U/mL.

### 2.7. Chitosan Hydrolysis

**Figure 3:**
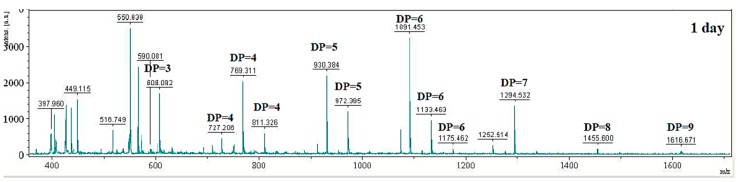
MALDI-TOF-MS spectrum of the chitooligomers (COS) obtained during chitosan hydrolysis with CS038. The proportion of low molecular weight oligomers was reduced by precipitation in the 90% methanol soluble/90% acetone insoluble fraction. The identified peaks are labelled with DP, in which DP indicates the degree of polymerization. The hydrolysis time is labelled in the spectrum.

To evaluate the applicability of CS038 for the enzymatic digestibility of chitosan into oligosaccharides, the crude enzyme from *B.* *mycoides* TKU038 was used in the experiments. Selective precipitation in 90% methanol and acetone solutions was performed to obtain low DP oligomers, as described earlier [5]. The enzyme hydrolyzed products of colloidal chitosan were analyzed by both high performance liquid chromatography (HPLC) (not shown) and matrix-assisted laser desorption ionization-time of flight (MALDI-TOF) mass spectrometry [19], as shown in Figure 3. The hydrolysate ions present in the mass spectra were identified as sodium adducts [M + Na^+^]. The peaks corresponding to the COSs with DP 3–9 were monitored in the spectrum, whereas monomers and dimers were not detected due to the interference of the matrix (below 500 *m*/*z*). After hydrolysis from the enzymatic reaction over six days, (GlcN)_2_–GlcNAc (*m*/*z* 566), GlcN–(GlcNAc)_2_ (*m*/*z* 608), (GlcN)_3_–GlcNAc (*m*/*z* 727), (GlcN)_2_–(GlcNAc)_2_ (*m*/*z* 769), (GlcN)_3_–(GlcNAc)_2_ (*m*/*z* 930), (GlcN)_2_–(GlcNAc)_3_ (*m*/*z* 972), (GlcN)_4_–(GlcNAc)_2_ (*m*/*z* 1091), and (GlcN)_3_–(GlcNAc)_3_ (*m*/*z* 1133) were the major products. In addition, other clear signals (*m*/*z* 659, 811, 1175, 1294, 1455, and 1616) were also detected (Figure 3). The peaks were [M + Na^+^] ion-peaks with a 161 Da mass larger than the peak ahead, which was exactly the molecular mass of a GlcN residue. The differences in *m*/*z* values of these signals with those of the corresponding COSs were 42 atomic mass units, which corresponded to the mass weight of an acetyl group. Thus, the peaks were [M + Na^+^] ion-peaks with a 203 Da mass larger than the peak ahead, which was the exact molecular mass of a GlcNAc residue. The hydrolysates contained chitooligomers (GlcN-oligomers) and several partial *N*-acetylated forms. Further, high-performance liquid chromatography (HPLC) analysis using Bond pack (NH_2_ column) showed that the hydrolysis products contained COS ranging from 1- to 9-mers, which indicated similar results as those shown by the MALDI-TOF MS spectrum. The TKU038 chitosanase reaction product is a mixture of DP 1–9 hetero-chitooligomers. These results indicate that CS038 might hydrolyse chitosan in an endo-type fashion. Based on these results, chitosan hydrolysis by CS038 combined with a selective methanol precipitation is a quick and simple method to obtain good chitooligosaccharide yields with up to nine DPs and low molecular weight oligomers. CS038 may be a useful tool for the industrial production of COSs and for research on the structure and biological functions of CS038 in nature.

### 2.8. DPPH Radical Scavenging Activity of COS

**Figure 4:**
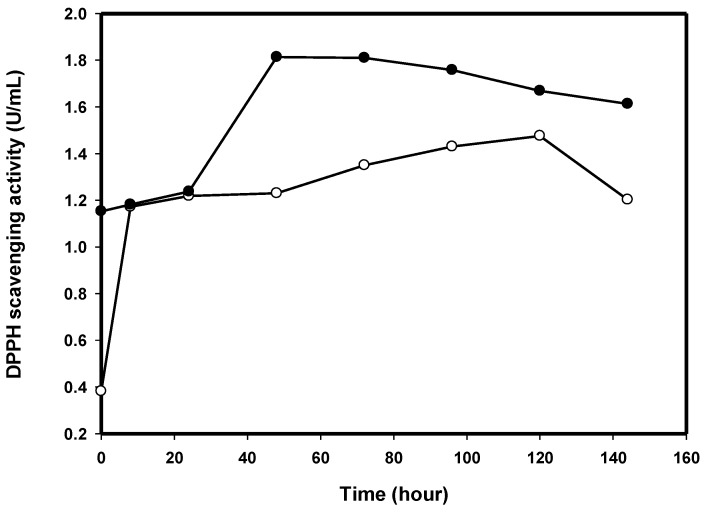
DPPH free radical scavenging activities of TKU038 culture supernatants (●) and CS038 hydrolysate (○) at various cultivation/reaction times.

Previous studies reported that COS, chitin, chitosan, and peptide exhibited high antioxidant activity [32,33,34,35,36,37,38,39,40,41] and anticarcinogenic properties [5,6,7]. In the culture supernatant of TKU038 chitosanase production, the reducing sugar content increased dramatically on the second day. In order to reutilize the reducing sugars efficiently, we incubated *B.* *mycoides* TKU038 for six days under the optimal culture conditions described above (0.5% SPP, 25 °C) and analyzed the antioxidant activity of the culture supernatant. The antioxidant activity assayed was the DPPH scavenging ability. The antioxidant activity (1.10 U/mL) was found in the supernatant of unfermented medium (day 0) and increased to 1.82 U/mL after fermenting with TKU038 for two days (Figure 4). We hypothesized that the autoclave treatment (121 °C for 15 min) degraded SPP and produced some antioxidant materials; however, some of the antioxidant materials were produced from the metabolism of strain TKU038. The differences in optimal culture time for chitosanase production (four days) and antioxidant production (two days) has demonstrated that the production of antioxidant materials might not be related to TKU038 chitosanase. The antioxidant compound in the TKU038 culture supernatant is worthy of further investigation.

On the other hand, the antioxidant activity of COS was also investigated. COSs were produced by the enzymatic hydrolysis of chitosan with 60% deacetylation from *B.* *mycoides* TKU038. After hydrolysis, the supernatant also showed antioxidant activity (Figure 4). Previous studies reported that COS exhibited high antioxidant activity, such as radical scavenging in vitro and inhibiting oxidative stress in cells. The antioxidant activity of COS was significantly related to the average molecular weight (*M*_W_) [42]. The effect of DP on DPPH radical scavenging activity was investigated further to identify the optimal DP range.

**Figure 5:**
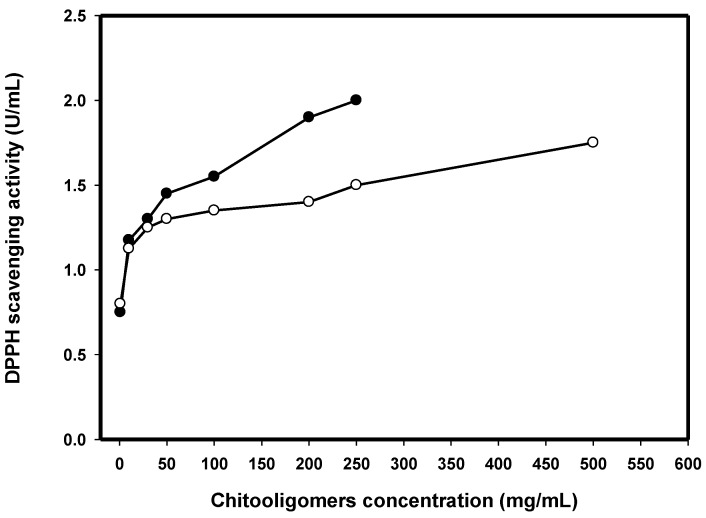
DPPH free radical scavenging activities of COSs hydrolyzed from CS038 with two types of degree of polymerization (DP) range (S1, 8 < DP < 16, (●); S2, DP < 8, (○)) at various concentrations.

The COS with different DP ranges were separated and lyophilized, and then, the antioxidant activity was investigated. The DPPH scavenging activities of COS with two types of DP ranges (S1, 8 < DP < 16 and S2, DP < 8) at different concentrations are presented in Figure 5. The two samples revealed apparent DPPH scavenging capabilities in a concentration-dependent fashion. The DPPH scavenging activity of S1 was higher than that of S2. The DPPH scavenging activity of S1 was 2 U/mL at 250 mg/mL. However, the DPPH scavenging activity of S2 was only 1.5 U/mL at 500 mg/mL. Similar results were reported by Li et al. [43] which showed the COS with DP 10–12 exhibited the strongest activity. Thus, we speculate that the COS with DP 11 played a major role in antioxidant activity. The TKU038 COS mixture with DP8-16 was more potent than the COS with DP < 8 in scavenging DPPH radical activity. These results confirmed that the COS with high DP (8 < DP < 16) would exhibit an enhanced ability to scavenge DPPH radicals when comparing to that with low DP (DP < 8).

### 2.9. Effect of COS on Cytotoxicity and Anti-Inflammation

NO is an extremely reactive free radical species that involved in numbers of pathological and physical processes. It plays a significant part in the pathophysiology of numerous diseases and its role in macrophage toxicity is also well studied. NO is recognized as a key pro-inflammatory mediator, which is involved in certain inflammatory disorders including chronic hepatitis, pulmonary fibrosis, and rheumatoid arthritis [44,45]. Although suitable levels of NO production are crucial in many normal physiological functions, a significant quantity of NO production could be cytotoxic leading to chronic inflammation, sepsis, and carcinogenesis [45,46,47,48,49].

**Figure 6:**
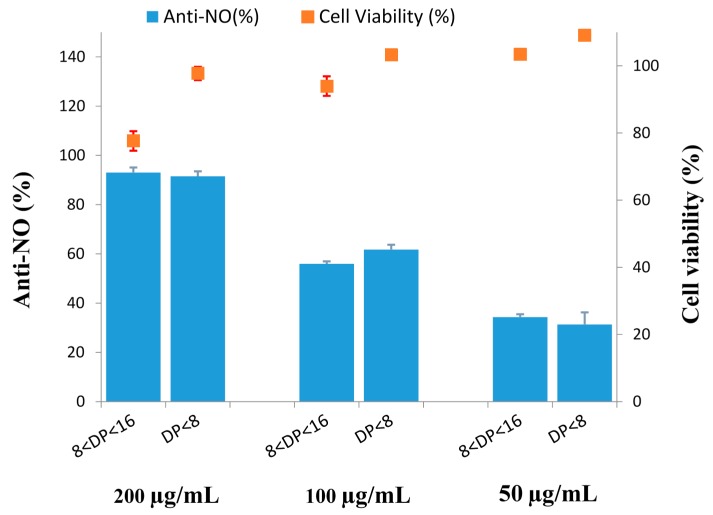
NO inhibitory activities of COSs hydrolyzed from CS038. Cell lines: The murine RAW 264.7 monocyte/macrophage cells. Cells were treated with LPS (1 µg/mL) or in combination with tested agents (200, 100, and 50 µg/mL) for 24 h.

In this study, the anti-inflammatory activity of COSs (S1 and S2) was estimated in vitro model with LPS-stimulated RAW 264.7 cells. The inhibition of LPS-stimulated NO secretion was due to the anti-inflammation. First, to examine the potential cell cytotoxicity by S1 and S2, MTT assay was conducted. When RAW 264.7 macrophages were treated with S1 and S2 at a concentration of 0, 50, 100, and 200 µg/mL, along with 1 µg/mL LPS, the resulting viabilities of RAW 264.7 cells were summarized in Figure 6. The results of statistical analysis indicated that treatment with S1 (8 < DP < 16) (50, 100 µg/mL) and S2 (DP < 8) (50, 100, 200 µg/mL) had no noticeable toxic effect on cell growth when comparing to 0.05% DMSO group (100.00% ± 2.17%). Although the viability of cells exposed to 200 µg/mL S1 was 77.64% ± 2.90%, it was significantly different from 0.05% DMSO group (Figure 6). S1 at a high concentration had the effect of cytotoxicity. These results revealed that no observable cytotoxicity was observed at concentration levels between 50 and 100 µg/mL of S1 and concentration levels between 50 and 200 µg/mL of S2. S1 and S2 (0–200 µg/mL) inhibited LPS-induced NO production in a concentration-dependent fashion. Although both S1 and S2 (200 µg/mL) were capable of inhibiting NO production by 93.07% ± 2.02% and 91.51% ± 1.99%, respectively in LPS-stimulated cells, S1 showed significant cytotoxicity. The IC_50_ values of S1 and S2 representing anti-inflammatory effect were of 82.65 ± 1.18 and 76.27 ± 1.49 µg/mL, respectively (Figure 6). These results indicated that S1 and S2 exhibited different degrees of anti-inflammatory capabilities. Anti-NO (%) was higher for S2 than for S1 at a concentration of 100 mg/mL, whereas the opposite behavior was observed for the other concentrations of 200 and 50 mg/mL. Take the error margin into consideration; we assume that the S2 might have higher activity than that of S1. Similar results were reported, as the low DP COS possessed higher anti-inflammatory effects, while the one with high DP COS possessed lower effects [43]. The findings of anti-inflammatory capability along with antioxidant activity of S2 make it a promising candidate for further investigations and could be recognized as a promising anti-inflammation agent based on its inhibitory profile on NO production.

Bioactive COS could have a substantial number of applications in biomedical and food industries. Chitosanase is the key enzyme that is required for the preparation of the bioactive COS from chitosan. Utilization of the squid by-products as the substrate for the production of chitosanase have commercial significance. Furthermore, our findings suggest that low DP COS could be the promising candidates for the development of potent anti-inflammatory agents.

### 2.10. Antitumoral Activities of COS

As damaging events are generally associated with oxidative stress, the prevalence of antioxidant and antitumor features in a single material would be highly desirable in terms of preventive, as well as therapeutic purposes. Hence, the cytotoxic activities of S1 and S2 were assessed in four tumoral cell lines (Hep G2, HEp-2, WiDr, and A549). Mitomycin-C was utilized as a positive control. In our preliminary experiments, the effects on Hep G2, HEp-2, WiDr, and A549 cell proliferation were measured via the MTT assay upon treatment with 80 µg/mL S1 and S2. The inhibition percentage of Hep G2, HEp-2, WiDr, and A549 were 7.53% ± 2.18%, 5.84% ± 1.91%, 7.66% ± 0.32%, and 6.75% ± 1.98%, respectively, after S1 treatment and 8.77% ± 1.28%, 6.99% ± 1.02%, 9.84% ± 3.64%, and 7.41% ± 0.95%, respectively, after S2 treatment. Similar results were also obtained in previous studies [5], in which 100 µg/mL of COS had no significant growth inhibition effects on CT26 cells. Since the 1980s, there has been a large number of research programs focusing on developing the antitumor activity of COS. Among them, DP6 COS was found to be able to suppress the growth of sarcoma 180 and MM-46 solid tumors transplanted in mice [43]. Comparing to the other single COSs (DP2, DP3, DP4, or DP5), DP6 COS showed a more potent inhibitory capability [43]. Comparable to the other reports, our studies revealed that low DP COS (DP < 8) possess slightly higher antitumoral activities than those of high DP COS (8 < DP < 16).

## 3. Experimental

### 3.1. Materials

Squid pens were acquired from Shin-Ma Frozen Food Co. (I-Lan, Taiwan). A water-soluble and low molecular weight chitosan (from 5 to 250 cps with minimal viscosity in water at 25 °C, 85% deacetylation degree, DD) from crab and shrimp shell waste was acquired from Charming and Beauty Co. (Taipei, Taiwan). Its average particle size was approximately 106 µm. Macro-prep DEAE was purchased from Bio-Rad. In addition, 2,2-diphenyl-1-picrylhydrazyl (DPPH) was bought from Sigma-Aldrich. Unless otherwise specified, all reagents used in this work were of the highest grade available.

### 3.2. Screening of Chitosanase-Producing Strains

The microorganisms were retrieved from soil samples, which were obtained at different locations in Taiwan. They were cultivated in SPP medium (pH 7.2) supplemented with 0.05% MgSO_4_·7H_2_O and 1% SPP, 0.1% K_2_HPO_4_ to screen for chitosan degrading activity. The strains were cultivated in a 250-mL Erlenmeyer flask that contains 50 mL of medium at 37 °C and 150 rpm for three days. The supernatants obtained by centrifugation were gathered for the determination of chitosanase activity using the protocol described in our previous paper [19]. Strain TKU038, which exhibited the highest activity, was selected for the further investigation.

### 3.3. Chitosanase Activity Assay

Chitosanase activity was assayed at 50 °C by the methods as described in our previous paper [9]. The reducing sugars released were determined with glucosamine as the reference compound to determine the enzyme activity [6].

### 3.4. Purification of Chitosanase

The chitosanolytic enzyme in the 768 mL cell free culture supernatant of *B.* *mycoides* TKU038 was concentrated with ammonium sulfate at 80% saturation, centrifuged at 12,000× *g* for 20 min to precipitate the enzyme, dissolved in a small amount of 50 mM sodium phosphate buffer (pH 7), and dialyzed using a 10-kDa molecular weight cut off membranes against 2 L of the same buffer for 24 h at 4 °C. The subsequent dialysate was charged onto a DEAE-Sepharose CL-6B column (5 cm × 30 cm) that had been pre-washed with the same phosphate buffer, and eluted with an isocratic gradient of 0.1 M NaCl-containing buffer. The fractions with high chitosanase activities were selected and concentrated by ammonium sulfate precipitation. After dialysis against the same phosphate buffer, the concentrates (5 mL) were charged on a Macro-prep DEAE column (12.6 mm × 40 mm). The chitosanase was eluted using an isocratic 0–1 M NaCl gradient in the same phosphate buffer. The chitosanase-active fractions were obtained and combined for subsequent characterization. Once the column chromatography operation was completed, the protein concentration was assessed by measuring the absorbance at 280 nm [6]. The concentration of the purified enzyme was determined based on the method reported by Bradford using bovine serum albumin as the standard. In addition, the molecular weight of the purified enzyme was determined by SDS-PAGE analysis.

### 3.5. Mass Spectrometry and Protein Identification

The band of attention was excised from the SDS-PAGE gel and identified by the same method as described in our previous paper [19].

### 3.6. Effects of pH and Temperature on Enzyme Activity and Stability

The optimum pH required for the relative chitosanase activities was determined at 50 °C using various pH buffers (pH value 4–11) at a 50 mM concentration [9]. The pH stability and thermal stability of CS038 were determined at pH 6 under the method as described in our published paper [9].

### 3.7. Kinetic Parameters

Various concentrations of water-soluble chitosan (0.005%–0.15% (*w*/*v*)) with a pre-determined enzyme concentration were prepared. Measurements were executed following the standard assay conditions under optimal conditions. The maximum velocity (*V*_max_) and Michaels-Mention constant (*K*_m_) were obtained from a Lineweaver-Burk plot.

### 3.8. Effects of Various Metal Ions on Chitosanase Activities

The influences of metal ions on the activities of CS038 were investigated as described in our previous paper [21]. The comparative activities were calculated under standard assay conditions, comparing to the one without metal ions and inhibitors (100%).

### 3.9. Enzymatic Production of the Chitosan Oligosaccharides

Chitosan (0.5% (*w*/*v*)) with 60% deacetylation was utilized as the substrate. The mixture of a TKU038 crude enzyme solution (1 mL) with chitosanase activity (5 U/mL) and substrate (1 mL) was incubated at 50 °C. Samples were withdrawn at 0 to 6 days from reaction mixtures for further preparation of the COS as described earlier [5].

### 3.10. Measurement of DPPH Radical Scavenging Activity

The diluted sample solution (150 µL) was mixed with 37.5 µL of a methanol solution containing 0.75 mM DPPH radical. The scavenging capability was estimated as described in our previous paper [39]. One unit of scavenging ability was expressed as the amount of sample that releases 50% scavenging activity under standard assay conditions.

### 3.11. Assay for Anti-Proliferation

The cytotoxicity of the tested sample was evaluated against HEp-2 (human laryngeal carcinoma), A549 (Human lung carcinoma), WiDr (human colon adenocarcinoma), and Hep G2 (human hepatocellular carcinoma) cell lines utilizing the MTT colorimetric protocol based on the well-established procedures [50]. The cells were cultured in MEM medium. After seeding cells in a 96-well microplate for 4 h, the 20 µL of sample was then placed in each well and incubated at 37 °C for additional 72 h. Then, 20 µL of MTT was added for 4 h. After washing off the medium and adding DMSO (200 µL/well) to the microplate with mechanical shaking for 30 min, the formazan crystals were re-dissolved and their absorbance was measured on a microtiter plate reader (Dynatech, MR 7000) at a wavelength of 550 nm. Mitomycin c (purity > 98%, Sigma-Aldrich) was utilized as a positive control.

## 4. Conclusions

In summary, we succeeded in developing an efficient production and purification procedure for an amphiprotic novel chitosanase (CS038) produced by *B.* *mycoides* TKU038 using an inexpensive medium based on squid pen powder. To the best of our knowledge, this may be the first report on chitosanase produced by *B.* *mycoides*. CS038 exhibited optimal pH values of 6 and 10 and broad range pH stability (4–10). The enzyme properties are advantageous for high activation under alkaline conditions, and these properties indicate potential applications in food industries and for waste treatment. Enzymatic hydrolysis by CS038 could lead to a large chitosan oligosaccharide with antioxidant activity, which was identified as an endo-chitosanase. Thus, this enzyme is an efficient tool for treating medical components and functional foods. The antioxidant activity of COS was strongly related to its DP. The COSs with a high DP exhibited enhanced DPPH radical scavenging compared with those with a low DP. Besides, our results demonstrated that different DP of COS hydrolyzed from CS038, especially S2 (DP < 8), was capable of inhibiting NO production. Our findings suggested that S2 might have a potential effect on the treatment with anti-inflammatory and antioxidant activities. The possible anti-inflammatory mechanism of S2 will be reported in detail in due course.

## Figures and Tables

**Table 1:** Purification summary of CS038 ^a^.

Step	Total			Specific Activity (U/mg)	Purification (Fold)	Recovery (%)
	Volume (mL)	Protein (mg)	Activity (U)			
Culture supernatant	768	2764.8	443.9	0.16	1	100
(NH_4_)_2_SO_4_ precipitation	45	751.5	386.0	0.51	3.19	87.0
DEAE-sepharose	40	128.2	261.4	2.04	12.75	58.9
Macro-Prep DEAE	10	7.5	156.1	20.81	130.06	35.2

^a^ *B.* *mycoides* TKU038 was grown in 50 mL of liquid medium in an Erlenmeyer flask (250 mL) containing 0.5% SPP, 0.1% K_2_HPO_4_, and 0.05% MgSO_4_·7H_2_O in a shaking incubator for four days at 25 °C.

**Table 2:** Comparison of CS038 with chitosanase from other microbes.

Strains	MW (kDa)	Optimal		Stability		Inhibitor	References
		Temp. (°C)	pH	Temp. (°C)	pH		
*B. mycoides* TKU038	48	50	6, 10	25–50	4–10	Cu^2+^, Ba^2+^, Zn^2+^, Fe^2+^, Mn^2+^, EDTA, PMSF	This study
*B. cereus* D-11	41	60	6	<50	5–10	Cu^2+^, Hg^2+^, Pb^2+^	[8]
*B. cereus* TKU022	44	60	7	25–40	7–10	Mn^2+^	[18]
*B. cereus* TKU031	43	50	5	20–50	5–9	Fe^2+^, Cu^2+^, Zn^2+^, Mn^2+^, EDTA	[19]
*B. cereus* TKU033	43	50	5	<40	5–7	Cu^2+^, Mn^2+^, EDTA	[20]
*B. cereus* TKU034	43	50	7	<50	4.5–7.5	Fe^2+^, Ca^2+^, Cu^2+^, Zn^2+^, Mn^2+^, EDTA	[21]
*Bacillus* sp. KCTC 0377BP	45	60	4–6	<55	4–8	Mn^2+^, Hg^2+^	[22]
*Bacillus* sp. TKU004	29	37	7	<40	4–7	Cu^2+^, Fe^2+^	[23]
*B. subtilis* TKU007	25	37	7	<37	4–9	Cu^2+^, Fe^2+^, EDTA	[24]
*B. subtilis* IMR-NK1	36	45	4	<40	5–9	Hg^2+^, PHMB	[25]
*Bacillus* sp. DAU101	27	50	7.5	-	-	Cu^2+^, Zn^2+^, Hg^2+^, Ni^2+^, Co^2+^	[26]
*Bacillus* sp. MET 1299	52	60	5.5	-	-	Mn^2+^, Cu^2+^, Zn^2+^, Co^2+^, EDTA	[27]
*B. criculans* MH-K1	32	50	6.5	-	-	Hg^2+^, Cd^2+^, Ni^2+^, Zn^2+^, pCMB	[28]
*Streptomyces griseus*	35	37	8	-	-	Ag^2+^, Hg^2+^, Fe^2+^, Cu^2+^, pCMB	[29]
*Streptomyces roseolus*	41	50	5	30–60	5–7	Mn^2+^, Cu^2+^, Zn^2+^, Co^2+^, EDTA	[17]
*Serratia* sp. TKU016	65	50	7	<50	6–7	Mn^2+^	[30]
*S. marcescens* TKU011	21	50	5	<50	4–8	Mn^2+^, Cu^2+^, PMSF	[12]
*Acinetobacter calcoaceticus* TKU024	66	60	7	<70	6–11	Mn^2+^, EDTA	[16]
	27	50	6	<90	4–10		

-: Not detected.

**Table 3:** Identification of CS038 by LC-MS/MS.

Peptide Sequence	Identified Protein and Coverage Rate	Accession Number
^81^SYYDNWKK^88^	Chitosanase 54%	*Bacillus cereus:* gi446936339
^93^NDLSSLPGGYYVKGEITGDADGFK		
PLGTSEGQGYGMIITVLMAGYDSNAQKIYDGLFK^150^		
^157^SSQNPNLMGWVVADSKKAQGHFDSATDGD		
LDIAYSLLLAHKQWGSNGTVNYLKEAKDMITK^217^		
^221^ASNVTNNNRLNLGDWDSKSSLD		
TRPSDWMMSHLRAFYEFTGDK^263^		
^283^YSPNTGLISDFVVKNPPQPAPKDFLEE		
SEYTNAYYYNASR^322^		
^327^IVMDYAMYGEK^337^		
^346^VSSWIQNK^353^		
^397^WVNSGWDWMK^406^		

**Table 4:** Effects of various chemicals on the activities of CS038.

Chemicals	Relative Activity (%)
None	100
Na^+^	94
Mg^2+^	93
Fe^2+^	0
Ca^2+^	88
Cu^2+^	21
Ba^2+^	57
Zn^2+^	20
Mn^2+^	0
EDTA	0
PMSF	0
